# Supplementary material for: Accumulation Dynamics of Defective Genomes during Experimental Evolution of Two Betacoronaviruses
Source: Viruses. 2024 Apr 20;16(4):644. doi: 10.3390/v16040644 (PMC11053736; doi:10.3390/v16040644)
Supplement: Supplementary file 1 [file viruses-16-00644-s001.zip › Table S1.pdf]

Table S1. Statistics describing the variation in MOI for each experimental evolution lineage. Median values ±IQR are shown. Last column shows the fold difference between high and low MOIs (averaging lineages).

**Virus**	**Cells**	**Lineage**	**Low MOI**	**High MOI**	**Log$_2$-fold**
HCoV-OC43	BHK-21	1	$3.74{\times}10^{-4}$ ±$2.50{\times}10^{-2}$	21.25 ±82.13	16.0
		2	$7.13{\times}10^{-4}$ ±$2.50{\times}10^{-2}$	31.25 ±208.50	
		3	$1.50{\times}10^{-4}$ ±$1.56{\times}10^{-2}$	25.00 ±145.75	
	HTC-8	1	$5.81{\times}10^{-5}$ ±$2.50{\times}10^{-3}$	0.88 ±4.76	12.6
		2	$1.21{\times}10^{-4}$ ±$3.37{\times}10^{-3}$	0.63 ±2.22	
		3	$3.00{\times}10^{-3}$ ±$2.96{\times}10^{-3}$	0.75 ±5.07	
MHV	CCL-9.1	1	$2.65{\times}10^{-6}$ ±$8.26{\times}10^{-6}$	$6.96{\times}10^{-3}$ ±0.28	12.0
		2	$2.59{\times}10^{-6}$ ±$1.79{\times}10^{-5}$	$1.06{\times}10^{-2}$ ±$9.02{\times}10^{-2}$	
		3	$8.69{\times}10^{-6}$ ±$1.10{\times}10^{-4}$	$1.09{\times}10^{-2}$ ±$6.62{\times}10^{-2}$	
