# Supplementary material for: Accumulation Dynamics of Defective Genomes during Experimental Evolution of Two Betacoronaviruses
Source: Viruses. 2024 Apr 20;16(4):644. doi: 10.3390/v16040644 (PMC11053736; doi:10.3390/v16040644)
Supplement: Supplementary file 1 [file viruses-16-00644-s001.zip › Table S2.pdf]

Table S2. Fitting of viral load data to the best-fitting ARIMA(*p, d, q*)[1] model based on the minimum BIC criterium. Errors represent ±1 SE.

Virus	Cells	MOI	Lineage	ARIMA	BIC	*Intercept*	Slope	Moving average	Autocorrelation 1	Autocorrelation 2
HCoV-OC43	BHK-21	High	1	0, 0, 1	64.54	8.717 ±0.143	0.015 ±0.005	0.319 ±0.118		
			2	1, 0, 0	142.25	11.779 ±0.386	0.025 ±0.013		0.394 ±0.138	
			3	0, 0, 0	232.22	18.415 ±0.612	0.041 ±0.022			
		Low	1	1, 0, 0	546.11		−0.820 ±1.228		0.821 ±0.076	
			2	2, 0, 1	143.65	13.336 ±0.465	0.017 ±0.016	0.565 ±0.161	−0.302 ±0.176	0.565 ±0.136
			3	2, 0, 0	243.91	18.850 ±1.352	0.043 ±41.346		0.268 ±0.268	0.284 ±0.146
	HCT-8	High	1	0, 0, 0	631.98		−48.459 ±41.346			
			2	0, 0, 0	104.77	14.737 ±0.365	−0.097 ±0.020			
			3	0, 0, 0	−22.13	6.895 ±0.050	−0.018 ±0.003			
		Low	1	0, 0, 1	640.54		−635.858 ±74.643	0.720 ±0.137		
			2	1, 0, 0	346.36		−6.764 ±0.807		0.462 ±0.462	
			3	2, 0, 0	374.66		−4.469 ±2.255		0.425 ±0.425	0.335 ±0.183
MHV	CCL-9.1	High	1	0, 0, 0	66.58	7.8304 ±0.367	−0.036 ±0.030			
			2	0, 0, 0	33.85	5.584 ±0.174	0.007 ±0.014			
			3	0, 0, 0	−15.11	3.793 ±0.002	0.057 ±0.005			
		Low	1	1, 0, 0	446.34		−789.109 ±330.142	0.781 ±0.130		
			2	1, 0, 0	446.46		−697.742 ±338.711	0.774 ±0.142		
			3	1, 0, 0	446.15		−866.648 ±317.089	0.779 ±0.125		

[1]ARIMA parameters: $p \geq 0$: order of the autoregressive model (number of time lags); $d \geq 0$: is the differencing order; $q \geq 0$: order of the moving-average model.
