# Supplementary figures and images for: Accumulation Dynamics of Defective Genomes during Experimental Evolution of Two Betacoronaviruses
Source: Viruses. 2024 Apr 20;16(4):644. doi: 10.3390/v16040644 (PMC11053736; doi:10.3390/v16040644)

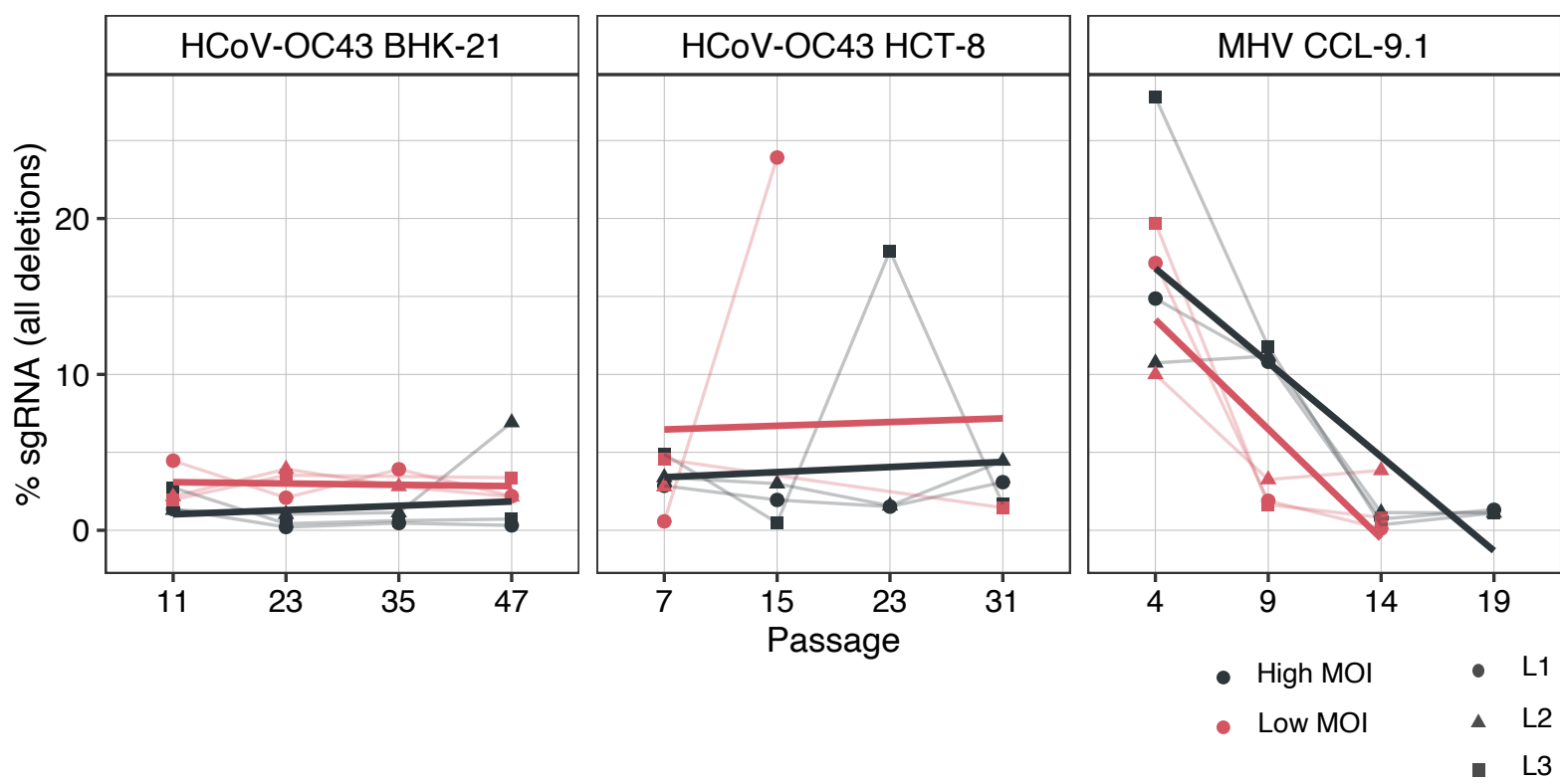

Supplement: Supplementary file 1 [file viruses-16-00644-s001.zip › Figure S1.pdf]

### HCoV-OC43 BHK-21

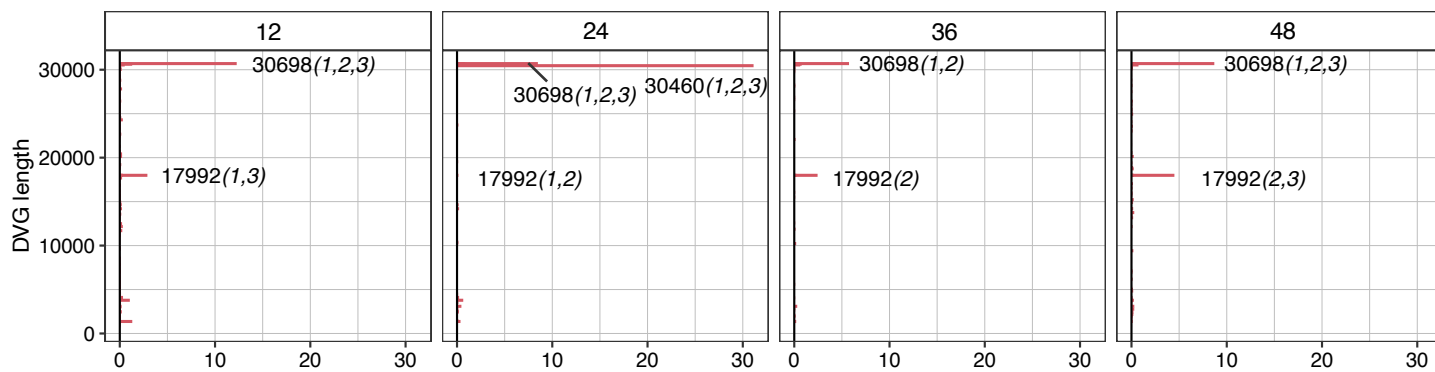

### HCoV-OC43 HCT-8

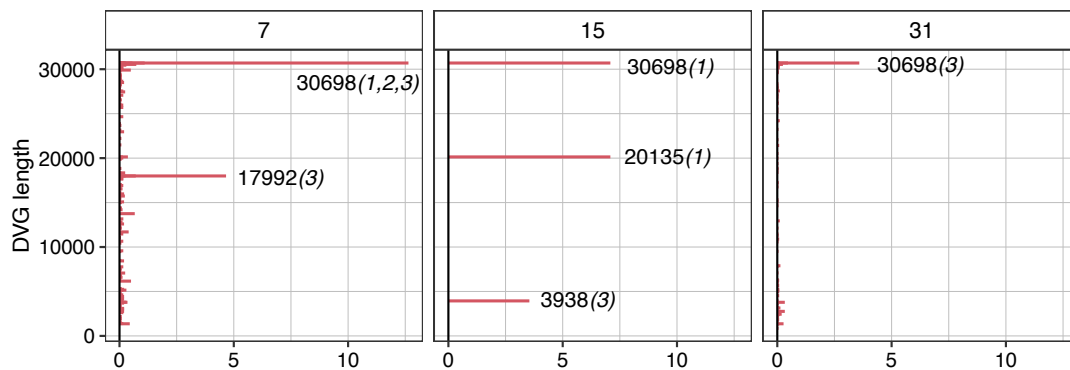

### MHV CCL-9.1

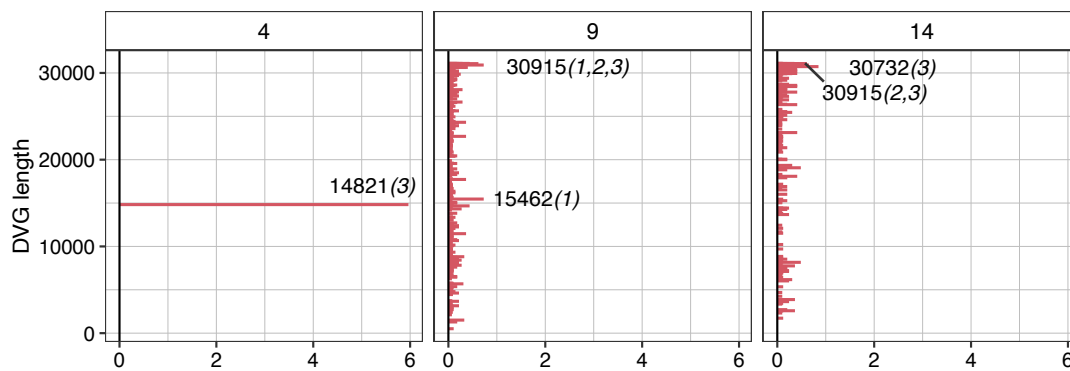

Abundance (sum RPHT)

Supplement: Supplementary file 1 [file viruses-16-00644-s001.zip › Figure S2.pdf]
